# Simulation-Guided Design and Synthesis of Functionalized Lactose-Crosslinked Degradable Molecularly Imprinted Polymer Nanoparticles for Sialic Acid Recognition

**DOI:** 10.3390/polym18172068

**Published:** 2026-08-26

**Authors:** Yining Li, Siqi Wang, Peifeng Li, Yinghan Zhao, Jin Chen, Ziyi Lin, Xintong Xu, Yi Ge

**Affiliations:** School of Pharmacy, Queen’s University Belfast, 97 Lisburn Road, Belfast BT9 7BL, UK; yli80@qub.ac.uk (Y.L.); swang54@qub.ac.uk (S.W.); pli07@qub.ac.uk (P.L.); yzhao32@qub.ac.uk (Y.Z.); jchen52@qub.ac.uk (J.C.); zlin08@qub.ac.uk (Z.L.); xxu23@qub.ac.uk (X.X.)

**Keywords:** molecularly imprinted polymer nanoparticles, sialic acid, degradable polymers, lactose-based crosslinker, pH-responsive hydrolysis, molecular docking

## Abstract

Aberrant cell-surface sialylation is widely associated with cancer progression and provides an accessible molecular feature for biosensing and targeted delivery. However, engineering molecularly imprinted polymer nanoparticles (nanoMIPs) that combine selective sialic acid (SA) recognition with controlled degradability and cytocompatibility remains challenging. In this study, a simulation-guided strategy was used to develop hydrolytically degradable SA-imprinted nanoMIPs incorporating a functionalized lactose-based crosslinker with cleavable ester linkages. Molecular docking and quantum-chemical calculations identified N-isopropylacrylamide (NIPAM), acrylamide (AAm), and N-hydroxyethyl acrylamide (HEAA) as complementary functional monomers and established an optimized SA:NIPAM:AAm:HEAA molar ratio of 1:1:2:1. The resulting nanoMIPs were spherical and nanoscale and exhibited pH-dependent hydrolytic mass loss that was more pronounced under mildly acidic conditions than at physiological pH. Compared with non-imprinted nanoparticles, the nanoMIPs displayed substantially enhanced SA binding, with a maximum binding capacity of 89.38 μmol g^−1^ and an imprinting factor of approximately 4.2, together with preferential recognition of SA over the selected competing molecules. MTT assays using MCF-7, HeLa, and HaCaT cells showed cell viability above 80% after 24 h exposure to 500 μg mL^−1^, indicating favorable short-term cytocompatibility. By integrating computationally optimized, multicomponent SA recognition with a carbohydrate-derived, hydrolytically degradable crosslinking strategy, this work addresses the coupled requirements of binding-site fidelity and material degradability within a single nanoMIP platform. These findings establish a materials-level foundation for future SA-directed biosensing and targeted delivery systems in cancer-relevant applications.

## 1. Introduction

Cancer remains a leading cause of morbidity and mortality worldwide, with approximately 20 million new cases reported annually [1]. Although chemotherapy remains indispensable in the management of many malignancies, its efficacy is frequently limited by systemic toxicity, nonspecific distribution, and inadequate drug accumulation at tumor sites [2]. Nanomedicine-based systems have therefore been developed to improve the stability, pharmacokinetics, and tissue distribution of anticancer agents through passive accumulation and active molecular recognition [3,4]. Liposomes [5], polymeric micelles [6], magnetic nanoparticles [7], mesoporous silica nanoparticles [8], and biodegradable polymeric systems [9] have all shown potential to improve therapeutic delivery. Nevertheless, favorable carrier properties and pharmacokinetics alone do not ensure tumor selectivity. Effective active-targeting systems also require recognition elements capable of engaging molecular features that are exposed, sufficiently abundant, and associated with malignant tissue in complex biological environments. Identifying such a target, while ensuring an acceptable material fate after administration, therefore remains a central challenge in cancer nanomedicine.

Molecularly imprinted polymers (MIPs), often described as synthetic antibody mimics, offer programmable molecular recognition together with physicochemical stability, synthetic flexibility, cost-effective production, and potentially high loading capacity [10,11,12]. These attributes have supported their investigation in biosensing [13], analytical separation [14], drug delivery [15], and cancer-oriented intracellular targeting [16]. MIPs are typically prepared by polymerizing functional monomers and crosslinkers around a molecular template; removal of the template creates binding cavities that are complementary to the target in size, shape, and chemical functionality. When prepared as nanoparticles, nanoMIPs additionally provide a high surface-area-to-volume ratio, readily accessible binding sites, and short diffusion pathways. Small, structurally defined molecules are particularly suitable imprinting templates because they generally exhibit less conformational variability than macromolecules such as proteins [17]. However, achieving selective rebinding in aqueous biological media remains difficult because template–monomer interactions must compete with solvation and nonspecific adsorption.

Sialic acids are a family of nine-carbon acidic monosaccharides located predominantly at the terminal positions of cell-surface glycoproteins and glycolipids. N-Acetylneuraminic acid (Neu5Ac), the predominant human sialic acid (SA) and the template investigated here, is particularly relevant to cancer because malignant transformation is frequently accompanied by increased or altered cell-surface sialylation. This remodeled glycocalyx has been associated with tumor-cell survival, invasion, metastatic dissemination, and evasion of antitumor immune responses [18]. The rationale for SA recognition is therefore twofold: terminal SA residues are directly accessible at the cell surface, and their aberrant density and presentation can provide a phenotypic handle for glycan profiling, cancer-cell recognition, and preferential engagement of diagnostic or delivery vehicles. SA is not exclusive to malignant cells, however; its cancer relevance depends on distinguishing disease-associated abundance and presentation rather than merely detecting its presence. Chemically, SA contains carboxyl, hydroxyl, and acetamido groups that can participate in complementary hydrogen-bonding and electrostatic interactions with functional monomers, making it amenable to molecular imprinting [18]. Previous SA- or monosaccharide-imprinted materials have enabled fluorescence-based cell recognition, cancer-cell imaging, and tumor-oriented delivery [19,20]. Nevertheless, free Neu5Ac binding does not by itself establish recognition of glycan-bound SA on living cells, and the simultaneous integration of selective recognition, controlled degradability, and cytocompatibility remains insufficiently addressed.

A further translational limitation is that conventional MIPs commonly rely on highly crosslinked, non-degradable polymer networks. Although extensive crosslinking preserves binding-cavity fidelity, persistent particles may restrict cargo release and raise concerns regarding long-term clearance and biological accumulation [15,21]. Previous studies have introduced carbohydrate-derived crosslinkers [22] and other degradable components [23,24] into MIP systems, but balancing structural stability with predictable degradation remains challenging: premature network cleavage may compromise recognition, whereas insufficient cleavage may negate the intended clearance or release advantage. Lactose is a hydroxyl-rich disaccharide that can be functionalized with polymerizable methacrylate groups, providing a carbohydrate-derived precursor for crosslinked polymer networks [25,26,27]. Such functionalization enables lactose to serve as a bio-based crosslinker while introducing hydrolyzable ester linkages into the polymer network [25,26]. Because ester cleavage is influenced by pH, local chemical environment, and water accessibility [28], this strategy offers a means of retaining the imprinted architecture during use while promoting subsequent hydrolytic breakdown. Nevertheless, lactose-crosslinked SA-imprinted nanoparticles, particularly systems whose recognition formulation is optimized computationally, remain underexplored.

Building on broader efforts to introduce degradability into MIP-based anticancer carriers [27], this study develops hydrolytically degradable nanoMIPs for SA recognition using a functionalized lactose-derived crosslinker containing cleavable ester linkages. Molecular docking and quantum-chemical calculations were combined to screen functional monomers, characterize template–monomer interactions, and optimize the prepolymerization formulation. On this basis, N-isopropylacrylamide (NIPAM), acrylamide (AAm), and N-hydroxyethyl acrylamide (HEAA) were selected as complementary functional monomers. The resulting nanoparticles were evaluated in terms of their physicochemical characteristics, SA-binding capacity, molecular selectivity, pH-dependent hydrolytic mass loss, and short-term in vitro cytocompatibility. The principal novelty is the integration of computationally guided, multicomponent SA recognition with a renewable, ester-containing lactose crosslinker within a single nanoscale platform, thereby addressing the competing requirements of binding-site fidelity and material degradability. This work establishes a materials-level foundation for the development of SA-directed biosensing and targeted delivery systems, warranting further investigation of cancer-cell recognition, in vivo biodistribution and clearance, and therapeutic efficacy.

## 2. Materials and Methods

### 2.1. Materials and Reagents

Anhydrous lactose was obtained from Glentham Life Sciences Ltd. (Corsham, UK). Methacryloyl chloride, methanol, acrylamide (AAm), pyruvic acid, 1,2-diamino-4,5-methylenedioxybenzene (DMB) and folic acid were purchased from VWR International Ltd. (Lutterworth, UK). Anhydrous acetonitrile, dimethyl sulfoxide-d_6_ (DMSO-d_6_, 99.8%) for nuclear magnetic resonance (NMR) analysis, acetic anhydride, pyridine, N-hydroxyethyl acrylamide (HEAA), N-isopropylacrylamide (NIPAM), dialysis membranes with a molecular-weight cut-off (MWCO) of 3500 Da, phenolphthalein, salicylic acid, and triethylamine were supplied by Merck Life Science UK Ltd. (Gillingham, Dorset, UK). N-Acetylneuraminic acid (sialic acid, SA) and 2,2′-azobis(2-methylpropionamidine) dihydrochloride (AAPH) were purchased from Fluorochem Ltd. (Hadfield, Derbyshire, UK). Unless otherwise stated, all reagents were used as received.

### 2.2. Computer Simulation

Molecular docking was performed using AutoDock 4.2.6 to investigate interactions between the SA template and the candidate functional monomers. AutoDock employs a semi-empirical free-energy force field incorporating van der Waals, hydrogen-bonding, electrostatic, desolvation, and torsional contributions, with conformational searching performed using the Lamarckian genetic algorithm. Solvent-related effects were accounted for through the desolvation term incorporated in the AutoDock scoring function. AutoDock uses 298.15 K as the reference temperature for estimation of inhibition constants. The resulting template–monomer binding conformations were visualized using PyMOL 2.5. Quantum-chemical calculations were subsequently conducted using GAMESS via the ChemCompute Science Gateway (https://chemcompute.org/, accessed on 18 August 2024) to evaluate the electronic properties and relative stabilities of the investigated template–monomer complexes. Geometry optimization was performed at the B3LYP/6-31G* level using a polarizable continuum model (PCM) with water as the solvent. Following geometry optimization, the minimum total electronic energies of each complex, the isolated template, and the corresponding functional monomers were calculated and expressed in atomic units (a.u.). The stabilization energy (ΔE) of each template–monomer complex was determined using Equation (1):

ΔE = E_Complex_ − (E_Template_ + ΣE_Monomer_) (1)

where E_Complex_, E_Template_ and E_Monomer_ represent the minimum total electronic energies of the optimized template–monomer complex, template molecule, and functional monomers, respectively.

### 2.3. Preparation of Hydrolytically Degradable MIP NPs

#### 2.3.1. Synthesis of the Lactose-Based Crosslinker

Anhydrous lactose (1 mmol) was suspended in dry acetonitrile (20 mL) in a round-bottom flask. A solution of methacryloyl chloride (12 mmol) and triethylamine (12 mmol) in dry acetonitrile (20 mL) was then added dropwise to the suspension at 0 °C. The reaction mixture was maintained at 0 °C for 2 h and subsequently stirred overnight at room temperature under a nitrogen atmosphere. Upon completion of the reaction, the mixture was centrifuged, and the supernatant containing unreacted methacryloyl chloride and soluble by-products was discarded. The resulting precipitate was washed three times with acetone to remove residual reagents and low-molecular-weight by-products. The purified lactose-based crosslinker was subsequently dried under vacuum at 40 °C for 2 h. After vacuum drying, the functionalized lactose derivative was obtained as a solid with an isolated yield of 69%.

#### 2.3.2. Synthesis of Hydrolytically Degradable MIP NPs

To form the prepolymerization complex, N-acetylneuraminic acid (SA; 0.2 mmol), the lactose-based crosslinker (150 mg), N-isopropylacrylamide (NIPAM; 0.2 mmol), acrylamide (AAm; 0.4 mmol), and N-hydroxyethyl acrylamide (HEAA; 0.2 mmol) were dispersed in 15 mL of an acetonitrile/deionized-water mixture (2:1, *v*/*v*). The mixture was shaken at room temperature for 1 h. Separately, 2,2′-azobis(2-methylpropionamidine) dihydrochloride (AAPH; 0.1 mmol) was dissolved in deionized water (3 mL) and sonicated for 10 min. The resulting initiator solution was added dropwise to the prepolymerization mixture. Polymerization was then conducted at 70 °C for 3 h under a nitrogen atmosphere with mechanical stirring. Non-imprinted polymer nanoparticles (NIP NPs) were prepared under otherwise identical conditions but without the addition of SA.

Following polymerization, the solvent was removed by rotary evaporation. The recovered nanoparticles were washed repeatedly with methanol/acetic acid (9:1, *v*/*v*) to extract the SA template. They were then washed sequentially with deionized water and acetone to remove residual acetic acid and other soluble impurities. The purified nanoparticles were dried under vacuum at 40 °C for 24 h.

### 2.4. Physicochemical Characterization

The hydrodynamic particle-size distribution and zeta potential were determined by dynamic light scattering (DLS) using a Zetasizer ZS instrument (Malvern Panalytical, Malvern, UK). Before analysis, the nanoparticles were dispersed in ultrapure water and sonicated for 15 min to obtain homogeneous suspensions. Both particle-size and zeta-potential measurements were performed in triplicate (n = 3). Ultraviolet–visible (UV–vis) absorption spectra were recorded using a Cary 60 double-beam UV–vis spectrophotometer (Agilent Technologies, Santa Clara, CA, USA). Particle morphology was examined by transmission electron microscopy (TEM) using a JEM-1400PLUS microscope (JEOL Ltd., Tokyo, Japan). For TEM analysis, the nanoparticles were dispersed in deionized water by sonication. A drop of each dispersion was deposited onto a copper grid and allowed to dry before imaging. Fourier-transform infrared (FTIR) spectra were acquired using a PerkinElmer Spectrum Two N spectrometer (PerkinElmer, Shelton, CT, USA). Nuclear magnetic resonance (NMR) spectra were recorded using a Bruker UltraShield spectrometer (Bruker, Rheinstetten, Germany) operating at 400 MHz, with DMSO-d_6_ as the solvent.

### 2.5. In Vitro pH-Dependent Hydrolytic Degradation Study

The pH-dependent hydrolytic degradation of the MIP nanoparticles was evaluated by monitoring changes in their dry mass over time. Briefly, MIP nanoparticles (15 mg) were placed in dialysis membranes with a molecular-weight cut-off (MWCO) of 3500 Da and immersed separately in phosphate-buffered saline (PBS) adjusted to pH 3.0, 6.0, 7.4, or 10.0. The samples were incubated at 37 °C in a thermostatically controlled water bath under continuous magnetic stirring.

At predetermined time points, the nanoparticles were recovered, rinsed thoroughly with deionized water, and dried. The remaining mass was calculated as (m_t_/m_0_) × 100%, where m_0_ is the initial dry mass of the nanoparticles and m_t_ is their dry mass after degradation for a specified period. Changes in nanoparticle morphology and particle size during degradation were assessed by transmission electron microscopy (TEM).

### 2.6. Equilibrium Binding Study

Sialic acid was quantified by derivatization with 1,2-diamino-4,5-methylenedioxybenzene (DMB), followed by fluorescence detection [29]. The DMB reagent contained 7 mM DMB, 1.4 M acetic acid, 0.75 M 2-mercaptoethanol, and 18 mM sodium hydrosulfite. For calibration, aliquots (400 μL) of SA standard solutions (10–400 μM) were mixed with an equal volume of DMB reagent and incubated at 50 °C for 150 min. The samples were then cooled on ice for 5 min and analyzed immediately by fluorescence spectroscopy using excitation and emission wavelengths of 373 and 448 nm, respectively.

For the equilibrium binding experiments, MIP or NIP nanoparticles (10 mg) were added to 5 mL of SA solution at initial concentrations ranging from 10 to 400 μM. The suspensions were sealed and gently agitated at room temperature for 2 h to reach binding equilibrium. The nanoparticles were subsequently separated by centrifugation at 4000 rpm for 10 min. An aliquot of each supernatant was mixed with an equal volume of DMB reagent, derivatized under the conditions described above, and analyzed by fluorescence spectroscopy. All measurements were performed in triplicate. The equilibrium binding capacity (Q) was calculated by mass balance using Equation (2):

(2) $$Q=\frac{\left(c_{i}-c_{s}\right)V}{m}$$

where Q (μmol g^−1^) is the equilibrium binding capacity, c_i_ and c_s_ (μmol L^−1^) are the initial and equilibrium concentrations of SA, respectively, V (L) is the volume of the binding solution, and m (g) is the mass of nanoparticles. The binding data were further analyzed using the Scatchard relationship given in Equation (3):

(3) $$\frac{Q}{c_{s}}=\frac{Q_{max}-Q}{K_{d}}$$

where Q_max_ (μmol g^−1^) is the maximum binding capacity and K_d_ (M) is the apparent dissociation constant. From the linear Scatchard plot, K_d_ was calculated as the negative reciprocal of the slope, and Q_max_ was calculated from the product of the y-intercept and K_d_. The imprinting factor (IF) was calculated using Equation (4):

(4) $$IF=\frac{Q_{MIP}}{Q_{NIP}}$$

where Q_MIP_ and Q_NIP_ are the equilibrium binding capacities of the MIP and NIP nanoparticles, respectively, measured under identical conditions.

### 2.7. Selectivity Study

The molecular selectivity of the MIP nanoparticles was evaluated using SA as the template analyte and folic acid, salicylic acid, and pyruvic acid as non-template comparators. MIP or NIP nanoparticles (5 mg) were added separately to 5 mL of each analyte solution (100 μM). The suspensions were sealed and incubated at room temperature for 2 h, after which the nanoparticles were separated by centrifugation at 4000 rpm for 10 min. The residual concentrations of folic acid and salicylic acid in the supernatants were determined by UV–vis spectroscopy at their respective maximum absorption wavelengths, whereas SA and pyruvic acid were quantified using the DMB derivatization method. The corresponding absorption spectra and calibration curves for the non-template analytes are provided in Figure S1. The equilibrium binding capacity and imprinting factor were calculated using Equations (2) and (4), respectively. All experiments were performed in triplicate.

### 2.8. Cell Culture and In Vitro Cytotoxicity

Human breast adenocarcinoma (MCF-7), cervical adenocarcinoma (HeLa), and immortalized human keratinocyte (HaCaT) cells were cultured in Dulbecco’s modified Eagle medium (DMEM) supplemented with 10% heat-inactivated fetal bovine serum (FBS) and 1% penicillin–streptomycin. The cells were maintained at 37 °C in a humidified atmosphere containing 5% CO_2_ and were routinely screened for mycoplasma contamination.

The cytotoxicity of the MIP and NIP nanoparticles was assessed using the MTT cell-viability assay. MCF-7, HeLa, and HaCaT cells were seeded in 96-well plates at a density of 1 × 10^4^ cells per well and incubated for 24 h at 37 °C in a humidified atmosphere containing 5% CO_2_. The culture medium was then replaced with fresh medium containing MIP or NIP nanoparticles at concentrations ranging from 0 to 500 μg mL^−1^, and the cells were incubated for a further 24 h. After treatment, 10 μL of MTT solution (5 mg mL^−1^) was added to each well, followed by incubation at 37 °C for 4 h to allow formazan formation. The medium was carefully removed, and the resulting formazan crystals were dissolved in DMSO (150 μL per well). Absorbance was measured at 570 nm using a microplate reader. Untreated cells and cell-free wells were used as controls and blanks, respectively. All experiments were performed in triplicate (n = 3), and cell viability was calculated using Equation (5) [12]:

(5) $$\mathrm{Cell}\;\mathrm{viability}\;(\%)\;=\frac{A_{sample}-A_{blank}}{A_{control}-A_{blank}}\times 100\%.$$

where A_sample_ is the absorbance of nanoparticle-treated cells, A_control_ is the absorbance of untreated cells, and A_blank_ is the absorbance of cell-free wells containing culture medium and MTT reagent.

## 3. Results and Discussion

### 3.1. Simulation-Guided Design of SA-Recognition Sites

**Figure 1:**
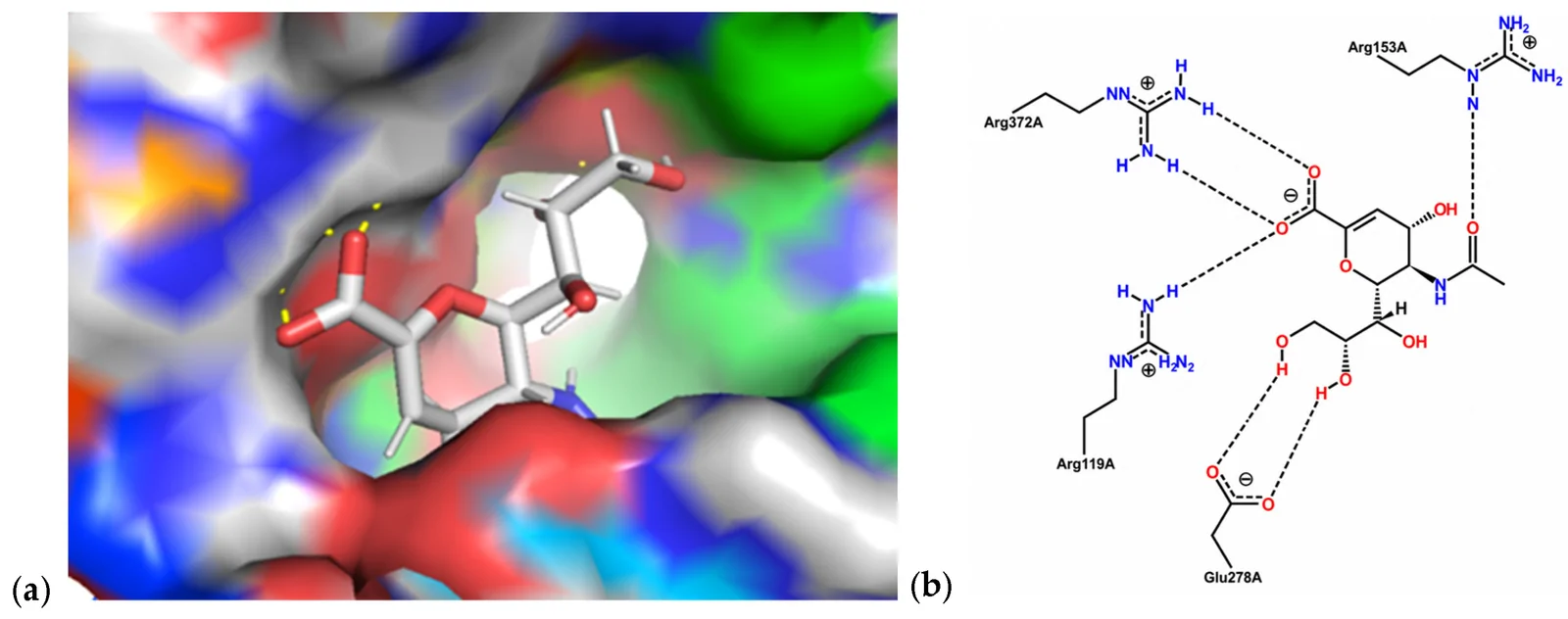
Analysis of SA-neuraminidase interactions used to identify candidate recognition groups: (**a**) optimized three-dimensional binding conformation visualized using PyMOL 2.5; and (**b**) two-dimensional interaction diagram generated using the Protein Plus SERVER (https://proteins.plus/, accessed on 14 August 2026).

The SA–neuraminidase complex was examined as a biological interaction model to identify chemically accessible recognition groups. As shown in Figure 1, the carboxyl, hydroxyl, and acetamido groups of SA participated predominantly in hydrogen-bonding contacts. Candidate monomers bearing complementary amide and hydroxyl functionalities were consequently prioritized for screening.

The predicted association of SA with the candidate functional monomers was evaluated using AutoDock, and the relative electronic stabilities of the optimized complexes were examined using the calculated ΔE values (Table 1). Within the AutoDock scoring framework, a more negative binding energy denotes a more favorable predicted association. The ΔE values provide a separate quantum-chemical comparison of the optimized complexes. Since both metrics are method-dependent and do not represent experimentally measured affinities, they were used comparatively rather than interpreted as absolute binding constants. Although the computational models account for solvent-related effects through the AutoDock desolvation term and the PCM water model used in the GAMESS calculations, they represent simplified molecular environments and do not fully reproduce the complexity of physiological media. In addition, the monomer selection was based on the combined consideration of both AutoDock binding energies and GAMESS stabilization energies (ΔE), rather than on the ranking of either parameter individually. Although N-phenylacrylamide showed slightly better docking than NIPAM, its greater hydrophobicity and steric bulk made it less suitable for the aqueous system. Likewise, despite its favorable ΔE, methacrylic acid was not selected because HEAA showed stronger docking and provided complementary hydroxyl and amide groups for SA recognition. Accordingly, NIPAM, AAm, and HEAA showed the most favorable combined profiles when predicted interaction energies, functional-group complementarity, and polymerization compatibility were considered, providing suitable interactions for organizing recognition sites around SA.

**Figure 2:**
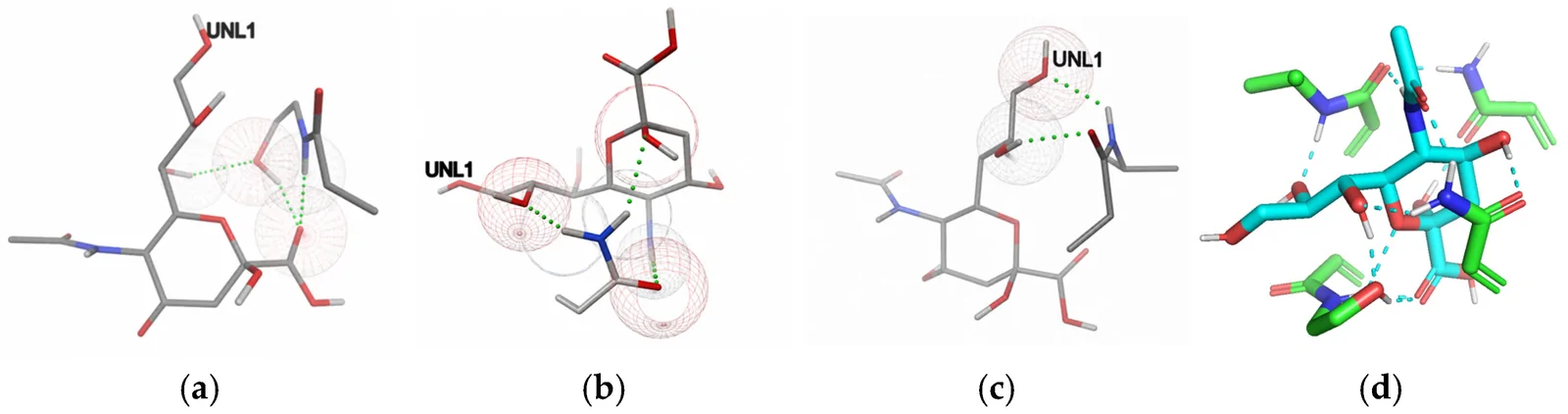
Predicted docking conformations of SA with (**a**) N-hydroxyethyl acrylamide (HEAA), (**b**) acrylamide (AAm), and (**c**) N-isopropylacrylamide (NIPAM), obtained using AutoDock 4.2; (**d**) optimized multiple-monomer conformation of the SA:NIPAM:AAm:HEAA complex at a molar ratio of 1:1:2:1, visualized using PyMOL.

Multiple-monomer docking was then used to examine how formulation stoichiometry affected the spatial organization of the prepolymerization complex. The SA:NIPAM:AAm:HEAA molar ratio of 1:1:2:1 produced the computationally favored arrangement, in which the monomers were distributed around the principal polar groups of SA (Figure 2). This multicomponent design was intended to create cooperative contacts that could better withstand competition from aqueous solvation than a single template-monomer interaction. Nevertheless, docking represents a static model and cannot reproduce solvent exchange, radical polymerization, crosslinking, or structural rearrangement during template removal. The experimental differences between MIP and NIP nanoparticles in the subsequent binding and selectivity studies therefore provide the critical test of whether the predicted interaction pattern translated into functional imprinted sites.

### 3.2. Synthesis and Physicochemical Characterization of Lactose-Crosslinked nanoMIPs

**Figure 3:**
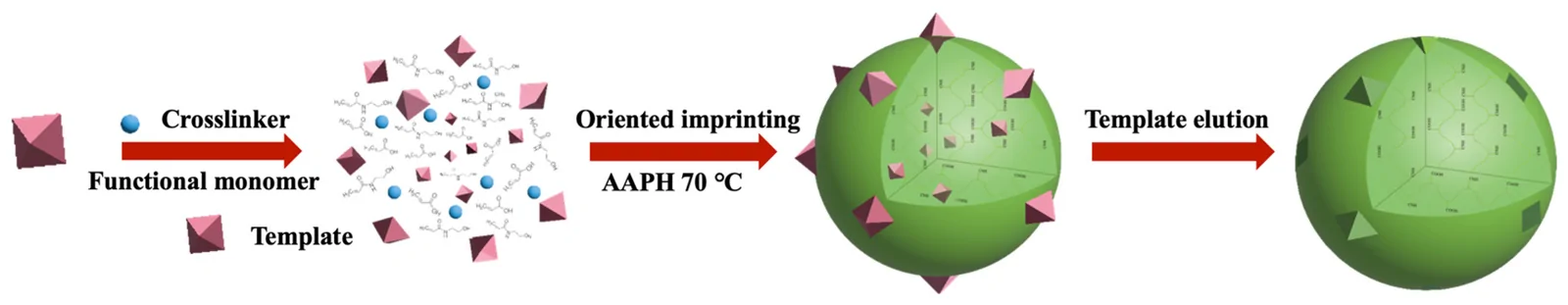
Schematic illustration of SA-imprinted nanoparticle synthesis and template removal.

The synthesis strategy translated the computationally selected recognition complex into a hydrolytically degradable nanoparticle network. As illustrated in Figure 3, SA was polymerized with HEAA, AAm, and NIPAM in the presence of the lactose-derived crosslinker. Subsequent template extraction generated cavities expected to reproduce the size and functional-group arrangement of SA. The resulting formulation therefore combined the simulation-selected recognition components with the hydrolysable lactose-derived crosslinked network.

**Figure 4:**
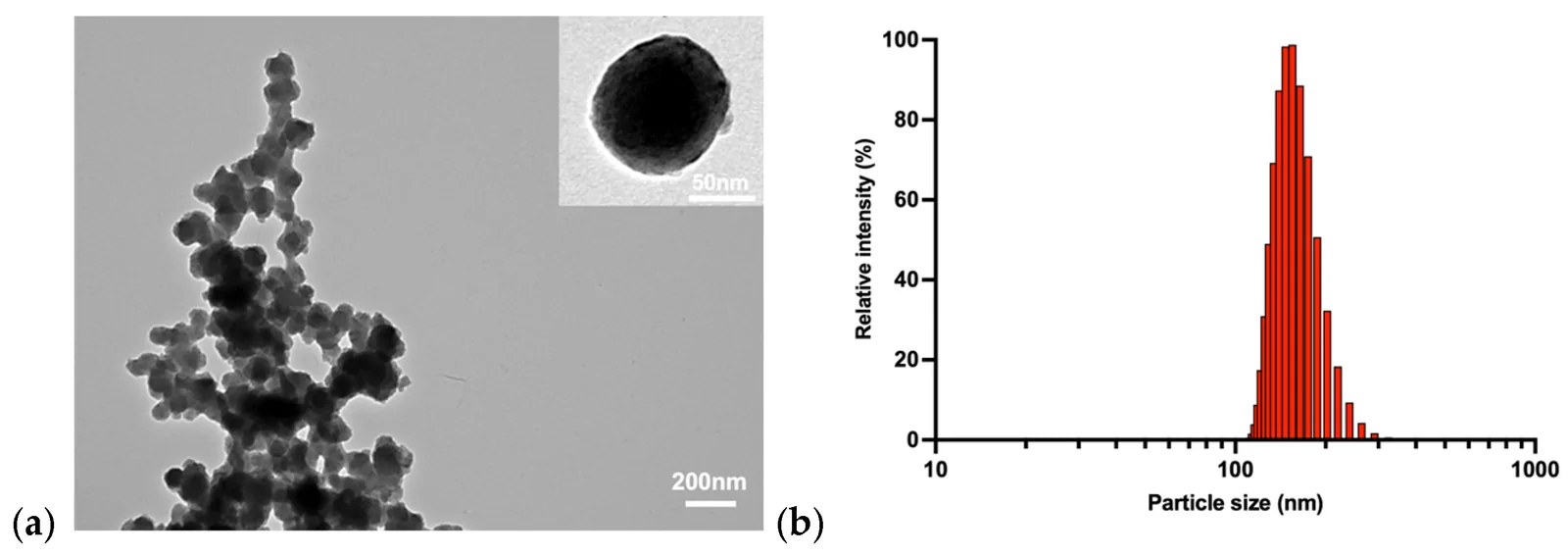
Physicochemical characterization of the SA-imprinted nanoparticles: (**a**) representative TEM image; and (**b**) hydrodynamic particle-size distribution measured by DLS.

TEM showed predominantly spherical nanoparticles with dry-state diameters of approximately 95–130 nm (Figure 4a). Some aggregation was apparent, plausibly reflecting drying and capillary forces during grid preparation. In aqueous dispersion, DLS yielded an average hydrodynamic diameter of 161 nm (n = 3) with a PDI of 0.21 ± 0.04 (Figure 4b), indicating a relatively narrow particle-size distribution. The larger DLS value is consistent with measurement of a hydrated and potentially swollen polymer layer, together with a possible contribution from small aggregates. The zeta potential was +3.91 ± 2.03 mV (mean ± SD, n = 3), indicating a near-neutral to slightly positive surface rather than a strongly cationic particle. This low magnitude suggests limited electrostatic stabilization and may partly explain the observed aggregation. Importantly, zeta potential alone does not establish enhanced cancer-cell adhesion or uptake. Those properties require direct assessment under biologically relevant ionic strength and serum conditions, including analysis of colloidal stability, protein-corona formation, and cellular internalization.

**Figure 5:**
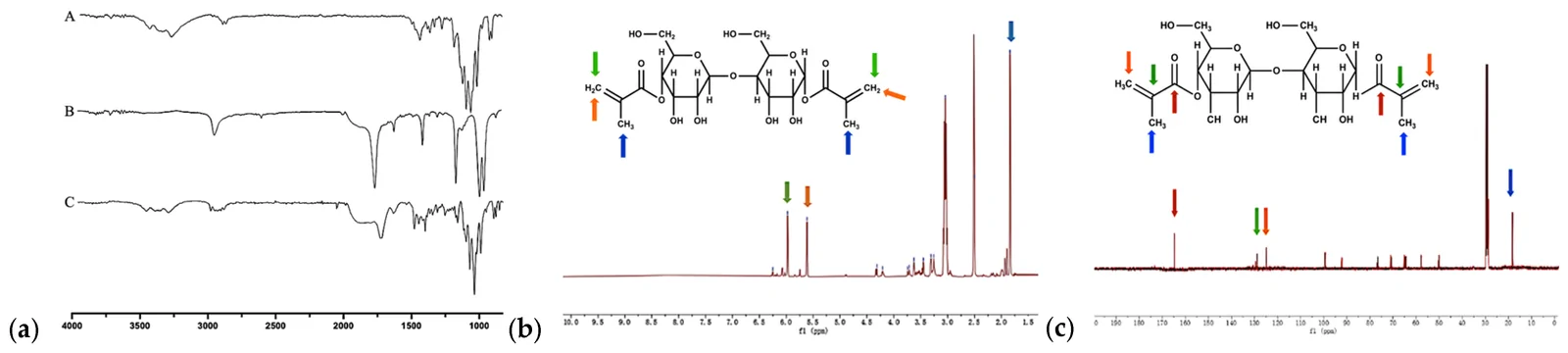
Structural characterization of the lactose-derived crosslinker: (**a**) FTIR spectra of (A) lactose, (B) the lactose-derived crosslinker, and (C) methacryloyl chloride; (**b**) ^1^H NMR spectrum; and (**c**) ^13^C NMR spectrum of the crosslinker in DMSO-d_6_.

The chemical structure of the lactose-derived crosslinker was first examined by FTIR spectroscopy. Lactose displayed a broad hydroxyl-stretching band at 3283 cm^−1^ and a characteristic C-O stretching band at 996.1 cm^−1^ (Figure 5a). Following reaction with methacryloyl chloride, the crosslinker spectrum exhibited a carbonyl band at 1724 cm^−1^ and a C=C-associated band at 1611 cm^−1^. The appearance of these bands supports the introduction of polymerizable methacrylate functionalities onto the lactose scaffold.

NMR spectroscopy confirmed the successful incorporation of methacrylate groups into the lactose-based crosslinker. In the ^1^H NMR spectrum (Figure 5b), signals at 1.83 ppm and 5.62/5.97 ppm were assigned to the methacrylate methyl and vinyl protons, respectively, and their approximate integral ratio of 3:1:1 was consistent with the methacrylate moiety. The ^13^C NMR spectrum (Figure 5c) contained signals at 17–18 ppm, 125–127 ppm, 130–132 ppm, and 164–165 ppm, attributable to the methyl, vinyl, substituted vinyl, and ester-carbonyl carbons, respectively. The degree of methacrylate substitution was determined by hydroxyl-group titration, which indicated a residual hydroxyl content of approximately 27%, corresponding to an estimated methacrylate substitution of 73%. Collectively, these orthogonal measurements support successful preparation of a highly functionalized, multifunctional lactose crosslinker. The high substitution level is expected to favor network formation, while the incorporated ester bonds provide the chemical basis for the hydrolytic behavior evaluated below.

### 3.3. pH-Dependent Hydrolytic Degradation and Network Stability

**Figure 6:**
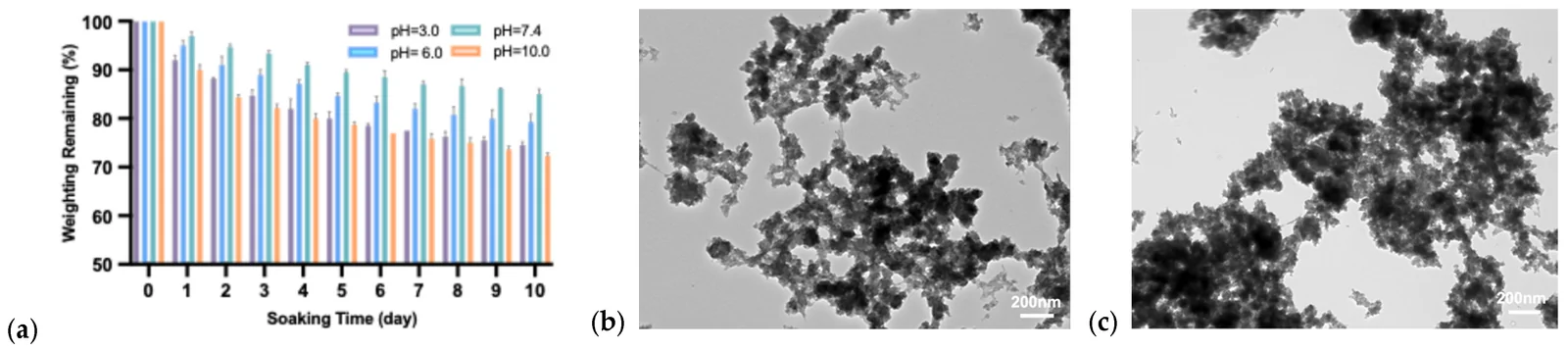
pH-dependent hydrolytic degradation of the MIP nanoparticles: (**a**) mass-loss profiles at 37 °C in PBS at pH 3.0, 6.0, 7.4, and 10.0; and representative TEM images after (**b**) 1 day and (**c**) 6 days of degradation. Data are presented as mean ± SD (n = 3).

The hydrolytic stability of the MIP nanoparticles was assessed at 37 °C in PBS at pH 3.0, 6.0, 7.4, and 10.0. The pH 7.4 condition approximated physiological buffer, whereas pH 6.0 provided a simplified mildly acidic condition relevant to, but not fully representative of, heterogeneous tumor extracellular environments. The pH 3.0 and 10.0 media served as accelerated acidic and alkaline stress conditions. As shown in Figure 6, mass loss was pH-dependent, reaching approximately 25.5%, 14.7%, and 27.7% after 240 h at pH 3.0, 7.4, and 10.0, respectively. At pH 6.0, the nanoparticles exhibited 21.3% mass loss after 240 h, indicating partial breakdown while retaining greater stability under physiological pH. These data support pH-modulated hydrolytic degradation but do not demonstrate complete degradation over the investigated period.

The most plausible mechanism is cleavage of the ester linkages introduced by the lactose-derived crosslinker, whose hydrolysis is influenced by pH and water accessibility [28]. Progressive cleavage would reduce network connectivity, promote swelling and fragmentation, and account for the morphological disruption observed by TEM. The network remained comparatively stable at pH 7.4 while becoming more labile under acidic conditions. Together, the gravimetric and TEM results support pH-dependent hydrolytic disruption of the ester-containing polymer network.

### 3.4. SA-Binding Performance

**Figure 7:**
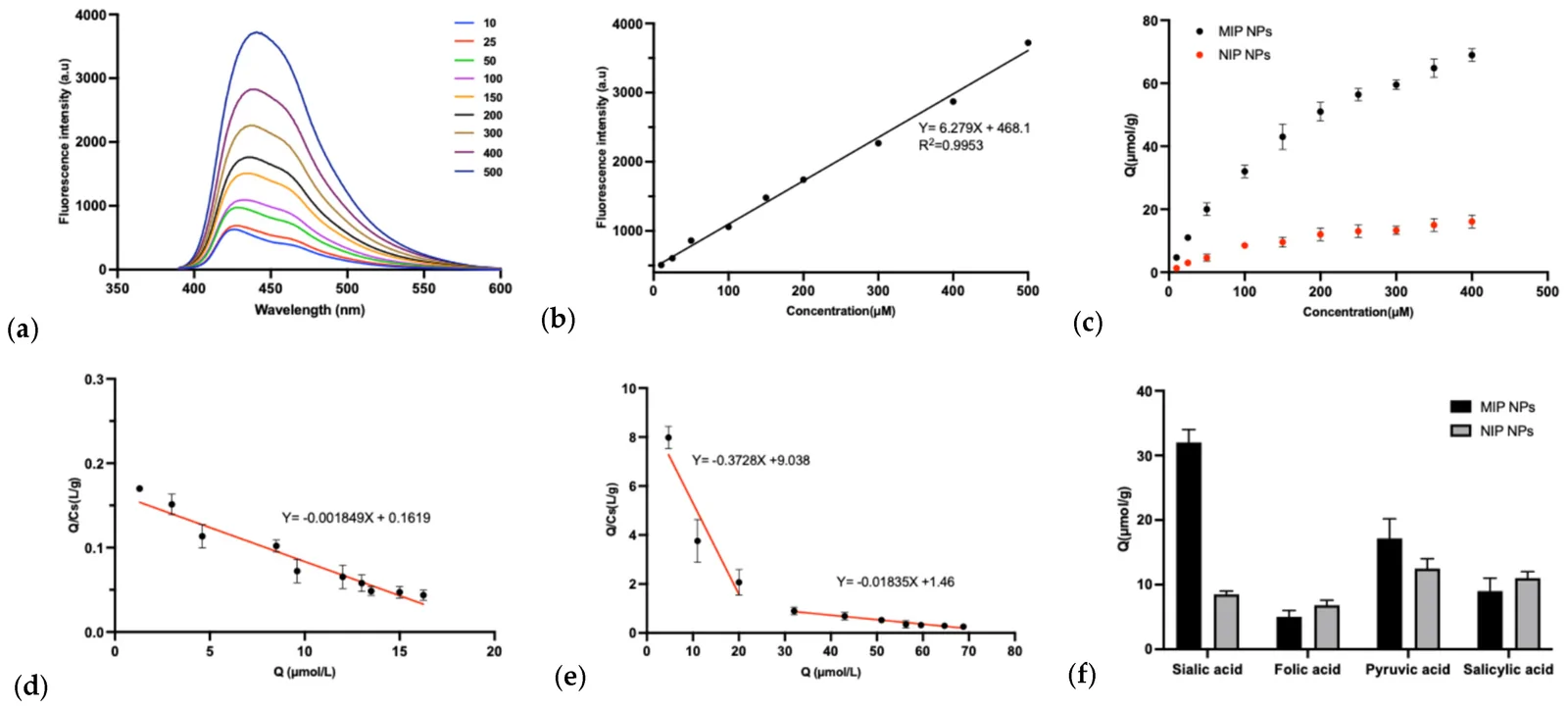
Binding and selectivity performance of MIP and NIP nanoparticles: (**a**) fluorescence emission spectra of DMB-derivatized SA standards (λ_ex_ = 373 nm); (**b**) SA calibration curve based on fluorescence intensity at 448 nm; (**c**) equilibrium SA-binding capacities at different initial concentrations; Scatchard plots for (**d**) MIP and (**e**) NIP nanoparticles; and (**f**) binding capacities toward SA, folic acid, pyruvic acid, and salicylic acid at an initial analyte concentration of 100 μM. Data are presented as mean ± SD (n = 3).

Equilibrium binding experiments were used to determine whether the simulation-guided formulation and imprinting process produced a measurable SA-recognition advantage over the compositionally matched NIP control. SA was quantified by DMB derivatization, and the calibration response was established over the investigated concentration range (Figure 7a,b). The binding capacity of both particle types increased with the initial SA concentration (Figure 7c), but the MIP nanoparticles showed consistently greater uptake than the NIP nanoparticles. The calculated Q_max_ values were 89.38 μmol g^−1^ for the MIP nanoparticles and 21.09 μmol g^−1^ for the NIP nanoparticles, based on the Scatchard analysis. The corresponding imprinting factor of approximately 4.2 demonstrates a substantial imprinting-dependent enhancement. Because the MIP and NIP formulations differed only by the presence of SA during polymerization, this comparison provides the clearest experimental evidence that template-directed cavities, rather than polymer composition alone, contributed to the enhanced binding.

The Scatchard analysis yielded two approximately linear regions for the MIP nanoparticles and one for the NIP nanoparticles (Figure 7d,e). The corresponding Scatchard-derived parameters are summarized in Table 2. The lower apparent K_d_ values of the MIP nanoparticles relative to the NIP nanoparticles are consistent with stronger SA binding after imprinting, while the two MIP regions suggest heterogeneous binding behavior with sites of different apparent affinities and capacities. The steeper region may reflect higher-affinity imprinted cavities, whereas the second region may include lower-affinity cavities and nonspecific matrix interactions. The single NIP region is consistent with a more uniform nonspecific binding contribution. These patterns support, but do not by themselves prove, the presence of two discrete classes of binding sites, because piecewise Scatchard interpretation is model dependent. Nonlinear isotherm fitting with uncertainty estimates would be valuable for a more rigorous mechanistic assignment of affinity and site heterogeneity.

The binding enhancement can be rationalized by cavities that combine steric complementarity with appropriately positioned amide and hydroxyl groups, allowing cooperative hydrogen-bonding and electrostatic interactions with SA. This interpretation is consistent with the simulation-guided design and the MIP-NIP binding difference. From a cancer-recognition perspective, however, these experiments establish selective rebinding of free Neu5Ac in solution, which is a necessary materials-level step but not evidence of recognition of glycan-bound SA on living cells. Previous SA- and monosaccharide-imprinted systems have demonstrated the potential of molecular imprinting for cell-surface recognition [19,20], supporting the future evaluation of the present nanoparticles in cancer-relevant cellular models.

### 3.5. Molecular Selectivity and Implications for Cancer-Associated SA Recognition

Molecular selectivity was evaluated using folic acid, pyruvic acid, and salicylic acid as non-template comparators with different sizes and functional-group arrangements. After incubation with the individual analytes, the MIP nanoparticles retained a markedly greater binding response toward SA than the NIP nanoparticles (Figure 7f). Binding of the three comparators was substantially lower, and their imprinting factors were 0.73 for folic acid, 1.36 for pyruvic acid, and 0.82 for salicylic acid, compared with 4.2 for SA. The slightly elevated IF observed for pyruvic acid (1.36) may reflect limited cross-recognition, potentially associated with its carboxyl functionality and partial structural similarity to SA. These results demonstrate preferential SA recognition within the tested panel and are consistent with binding sites that require an appropriate combination of molecular size, shape, and functional-group positioning rather than nonspecific adsorption alone.

The comparator panel provides an initial test of chemical discrimination, but it does not reproduce the complexity of a biological glycocalyx. The analytes are structurally distinct from SA, the measurements were conducted in single-analyte solutions, and no closely related sialic-acid derivatives, competing saccharides, proteins, or serum components were examined. Moreover, SA is present on healthy as well as malignant cells; cancer-associated recognition would depend on preferential engagement of altered SA density and glycan presentation rather than SA binding alone. Future validation should therefore include mixed-analyte competition, structurally related glycans, glycan-bound SA, desialylated controls, and comparisons between cell models with quantified differences in surface sialylation.

As summarized in Table 3, several strategies, including biodegradable, thermo-responsive, photoresponsive, and pH-responsive designs, have been developed to improve conventional MIPs. Although some reported systems exhibit higher IF or binding capacities, many remain non-degradable. In comparison, the present SA-imprinted nanoMIPs combine nanoscale dimensions, a Q_max_ of 89.38 μmol g^−1^ (27.64 mg g^−1^), an IF of approximately 4.2, and measurable hydrolytic degradation. The main advantage of this system therefore lies in integrating selective SA recognition with a degradable lactose-derived polymer network.

### 3.6. In Vitro Cytocompatibility

**Figure 8:**
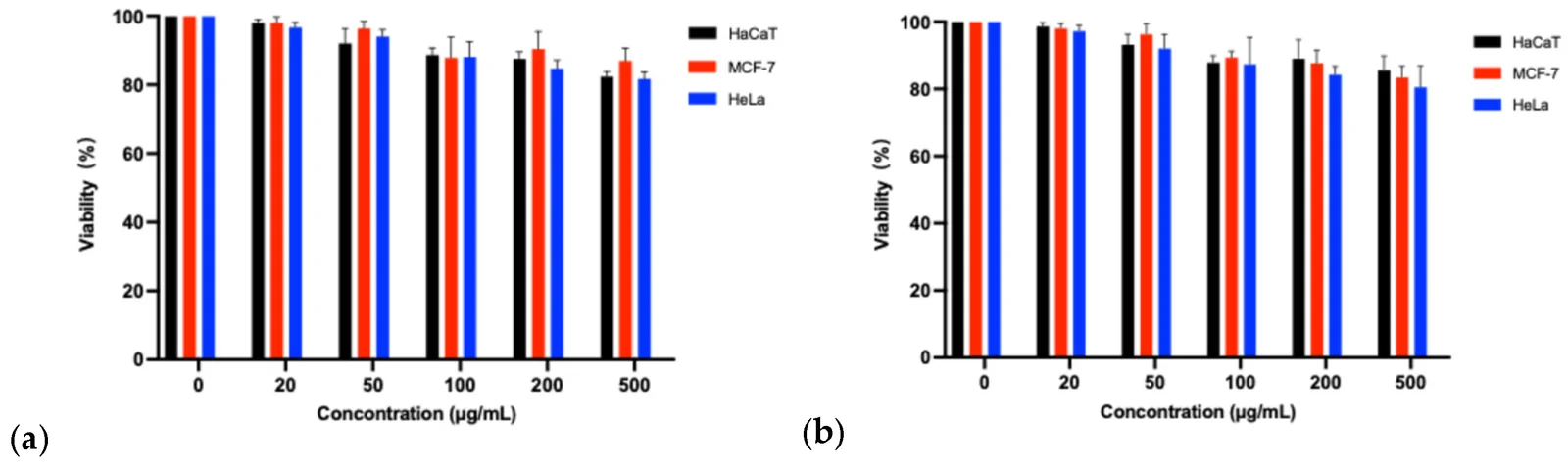
Cell viability of MCF-7, HaCaT, and HeLa cells after 24 h exposure to (**a**) MIP nanoparticles and (**b**) NIP nanoparticles. Data are presented as mean ± SD (n = 3).

Short-term in vitro cytocompatibility was evaluated by MTT assay after 24 h exposure of MCF-7, HeLa, and HaCaT cells to MIP or NIP nanoparticles at concentrations of 50–500 μg mL^−1^. As shown in Figure 8, both formulations produced only a modest concentration-dependent reduction in cell viability. At 500 μg mL^−1^, the viabilities of HaCaT, MCF-7, and HeLa cells treated with MIP nanoparticles were approximately 83%, 86%, and 81%, respectively, and the corresponding NIP-treated cultures showed comparable responses. These results indicate favorable short-term cytocompatibility of the nanoparticles under the tested conditions.

The similar viability profiles observed across the cancer and non-malignant cell lines indicate no marked cell-line-dependent acute toxicity. However, the present cytotoxicity experiments were designed to evaluate material compatibility and do not establish SA-mediated cellular recognition or selective uptake. Demonstration of cellular targeting will require dedicated studies using cell models with defined differences in surface sialylation, together with quantitative binding or uptake and appropriate competition or blocking controls. Taken together, the results demonstrate that the lactose-crosslinked nanoMIPs combine imprinting-dependent SA recognition, pH-dependent hydrolytic degradation, and favorable short-term cytocompatibility within a single nanoscale platform.

## 4. Conclusions

In this study, hydrolytically degradable molecularly imprinted polymer nanoparticles were developed for the selective recognition of sialic acid (SA) using a functionalized lactose-based crosslinker containing cleavable ester linkages. Molecular docking and quantum-chemical calculations guided the monomer selection and identified an optimal SA:NIPAM:AAm:HEAA molar ratio of 1:1:2:1. Structural analyses confirmed successful crosslinker synthesis, with approximately 73% of the lactose hydroxyl groups functionalized with methacrylate moieties. The resulting nanoMIPs exhibited a spherical nanoscale morphology and a slightly positive zeta potential of +3.91 ± 2.03 mV. Importantly, the nanoparticles combined imprinting-dependent SA recognition with pH-responsive hydrolytic degradation. They achieved a maximum binding capacity of 89.38 μmol g^−1^, an imprinting factor of approximately 4.2, and preferential SA binding over the selected competing molecules, while exhibiting enhanced hydrolytic mass loss under mildly acidic conditions compared with physiological pH. Furthermore, cell viability remained above 80% in MCF-7, HeLa, and HaCaT cells following 24 h exposure to nanoparticle concentrations of up to 500 μg mL^−1^, indicating favorable short-term cytocompatibility. Collectively, this work demonstrates that selective molecular recognition and hydrolytic degradability can be integrated within a single carbohydrate-crosslinked nanoMIP platform. These findings establish a materials-level foundation for the future development of SA-directed biosensing and targeted delivery systems, including applications targeting cancer-associated cell-surface sialylation, subject to further validation in complex biological and disease-relevant models.

## Figures and Tables

**Table 1:** Predicted AutoDock binding energies and calculated quantum-chemical ΔE values for complexes of SA with the candidate functional monomers.

Functional Monomer	Binding Energy (kcal mol^−1^)	ΔE (a.u.)
Methacrylic acid	−2.25	16.2767
N,N′-methylene bisacrylamide	−1.31	19.8206
N-isopropylacrylamide	−2.40	18.047
2-(dimethylamino)ethyl methacrylate	−1.07	23.636
N-phenylacrylamide	−2.46	19.136
N-hydroxyethyl acrylamide	−3.17	18.517
2-aminoethyl methacrylate hydrochloride	−1.77	18.845
Acrylamide	−2.61	14.034

**Table 2:** Scatchard binding parameters for SA recognition by MIP and NIP nanoparticles.

Sample/Region	Apparent K_d_ (M)	Apparent Q_max_ (μmol g^−1^)	R^2^
MIP Region I	4.22 × 10^−6^	32.43	0.822
MIP Region II	5.95 × 10^−5^	89.38	0.916
NIP	1.18 × 10^−4^	21.09	0.948

**Table 3:** Comparison of representative optimized molecularly imprinted polymer systems.

Template	Functional Mechanism	Size (nm)	Q_max_ (mg g^−1^)	K_d_/Affinity	IF	Degradation/Responsiveness	Ref
Sialic acid	Fluorescent surface imprinting/boronate affinity	~50	NR	K_d_ = 2.0 × 10^−4^ M	8.4	NR	[20]
Berberine chloride	Thermo-responsive adsorption/release	NR	33.44	NR	4.71	Reversible thermo-responsive adsorption/release over 25–45 °C	[30]
Sialic acid	Fluorescent core–shell MIP particles	~188	NR	K_a_^fluo^ = 8.87 × 10^4^ M^−1^	2.00–2.35	NR	[18]
Sulfamethazine	Photoresponsive azobenzene isomerization	~250	12.5	NR	3.01	Reversible light-controlled adsorption/release	[31]
Matrine	pH-responsive drug delivery	NR	21.48	NR	2.36	pH-responsive release	[32]
Sulfamethazine	Photoresponsive magnetic MIP/azobenzene isomerization	~340	25.06	NR	2.09	Reversible 365/440 nm light-controlled uptake/release	[33]
Docetaxel	Biodegradable glucose-based magnetic MIP/targeted drug delivery	~250	72	NR	NR	Biodegradable glucose-based crosslinked network	[27]

## Data Availability

All the data relevant to this study are provided in tables and figures. Further information can be requested from the corresponding author.

## Supplementary Materials

The following supporting information can be downloaded at: https://www.mdpi.com/article/10.3390/polym18172068/s1, Figure S1: (a) UV–vis absorption spectrum of folic acid. (b) Calibration curve of folic acid based on the absorbance at 280 nm. (c) UV–vis absorption spectrum of salicylic acid. (d) Calibration curve of salicylic acid based on the absorbance at 295 nm. (e) Calibration curve of pyruvic acid based on fluorescence intensity at 448 nm.

## Abbreviations

The following abbreviations are used in this manuscript:

AAm	Acrylamide
AAPH	2,2′-azobis(2-methylpropionamidine) dihydrochloride
ACN	Acetonitrile
DLS	Dynamic light scattering
DMB	1,2-diamino-4,5-methylenedioxybenzene
DMEM	Dulbecco’s Modified Eagle Medium
DMSO	Dimethyl sulfoxide
FBS	Fetal bovine serum
FTIR	Fourier-transform infrared spectroscopy
HEAA	N-hydroxyethyl acrylamide
IF	Imprinting factor
Kd	Dissociation constant
MIP	Molecularly imprinted polymer
MIP NPs	Molecularly imprinted polymer nanoparticles
MTT	3-(4,5-Dimethylthiazol-2-yl)-2,5-diphenyltetrazolium bromide
MWCO	Molecular weight cut-off
NIP	Non-imprinted polymer
NIP NPs	Non-imprinted polymer nanoparticles
NIPAM	N-isopropylacrylamide
NMR	Nuclear magnetic resonance
NPs	Nanoparticles
PBS	Phosphate-buffered saline
SA	Sialic acid
SD	Standard deviation
TEM	Transmission electron microscopy
UV–vis	Ultraviolet–visible spectroscopy
