# Supplementary material for: Simulation-Guided Design and Synthesis of Functionalized Lactose-Crosslinked Degradable Molecularly Imprinted Polymer Nanoparticles for Sialic Acid Recognition
Source: Polymers (Basel). 2026 Aug 26;18(17):2068. doi: 10.3390/polym18172068 (PMC13568074; doi:10.3390/polym18172068)
Supplement: Supplementary file 1 [file polymers-18-02068-s001.zip › polymers-4482962-supplementary.pdf]

Supporting Information

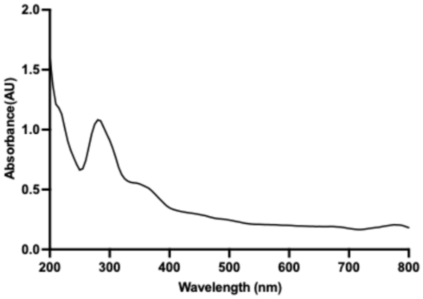

(a)

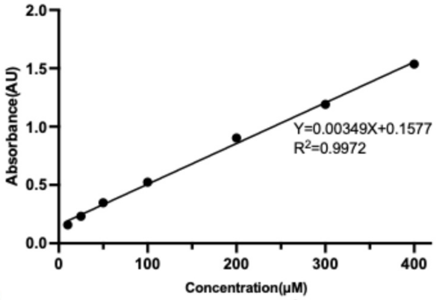

(b)

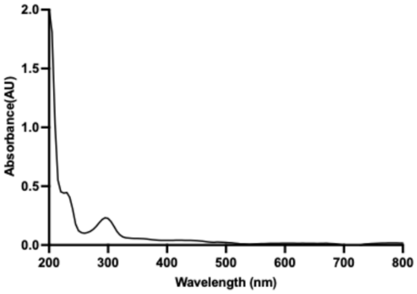

(c)

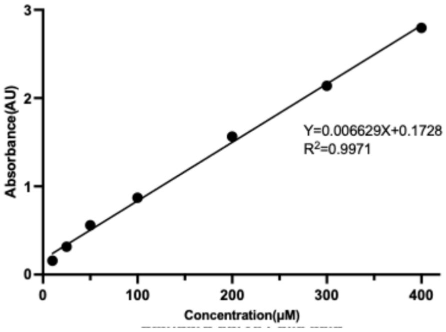

(d)

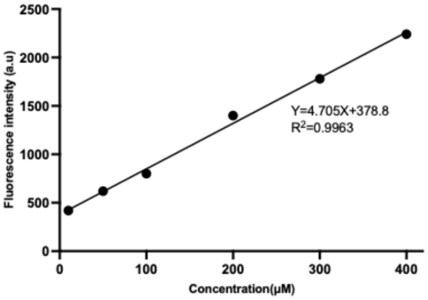

(e)

Figure S1. (a) UV–vis absorption spectrum of folic acid. (b) Calibration curve of folic acid based on the absorbance at 280 nm. (c) UV–vis absorption spectrum of salicylic acid. (d) Calibration curve of salicylic acid based on the absorbance at 295 nm. (e) Calibration curve of pyruvic acid based on fluorescence intensity at 448 nm.
